# The genome sequence of lesser burdock, *Arctium minus* (Hill) Bernh. (Asteraceae)

**DOI:** 10.12688/wellcomeopenres.23160.1

**Published:** 2024-10-15

**Authors:** Maarten J. M. Christenhusz, Claudia A. Martin, Royal Botanic Gardens Kew Genome Acquisition Lab, Plant Genome Sizing collective, Darwin Tree of Life Barcoding collective, Wellcome Sanger Institute Tree of Life Management, Samples and Laboratory team, Wellcome Sanger Institute Scientific Operations: Sequencing Operations, Wellcome Sanger Institute Tree of Life Core Informatics team, Tree of Life Core Informatics collective, Darwin Tree of Life Consortium

**Affiliations:** 1 Curtin University, Perth, Western Australia, Australia; 2 Hortus Botanicus, University of Technology Delft, Delft, The Netherlands; 3 Royal Botanic Garden Edinburgh Library, Edinburgh, Scotland, UK; 4 The University of Edinburgh, Edinburgh, Scotland, UK

**Keywords:** Arctium minus, lesser burdock, genome sequence, chromosomal, Asterales

## Abstract

We present a genome assembly of a diploid specimen of *Arctium minus* (lesser burdock; Tracheophyta; Magnoliopsida; Asterales; Asteraceae). The genome sequence is 1,903.1 megabases in span. Most of the assembly is scaffolded into 18 chromosomal pseudomolecules. The mitochondrial and plastid genome assemblies have lengths of 312.58 kilobases and 152.71 kilobases, respectively. Gene annotation of this assembly on Ensembl identified 27,734 protein-coding genes.

## Species taxonomy

Eukaryota; Viridiplantae; Streptophyta; Streptophytina; Embryophyta; Tracheophyta; Euphyllophyta; Spermatophyta; Magnoliopsida; Mesangiospermae; eudicotyledons; Gunneridae; Pentapetalae; asterids; campanulids; Asterales; Asteraceae; Carduoideae; Cardueae; Arctiinae; *Arctium*; *Arctium minus* (Hill) Bernh., 1800 (NCBI:txid143172).

## Background

Lesser burdock (also known as little burdock, louse-bur, common burdock, button-bur, cuckoo-button, or wild rhubarb), of the genus *Arctium* L., is a biennial herbaceous plant belonging to the daisy family, Asteraceae. This species, commonly known for its burr-like seed heads, is widespread and abundant across most of Britain where it is commonly perceived as a persistent weed ( Preston *et al.*, 2002). Its distribution ranges across lowland and upland landscapes, although it is less common in the far north and west of Scotland. The adaptability of *A. minus* to various soil types and climatic conditions has facilitated its spread and establishment in diverse geographic regions, and it thrives in a variety of urban and rural environments. It is particularly successful in areas with disturbed habitats such as roadsides, field margins, woodland edges and waste grounds, where there is minimal competition with other plant species. *A. minus* is native to all of Europe and western Asia, extending south to Morocco and east to Afghanistan, and it has been widely introduced across North America, southeastern Brazil, southeastern Australia and the North Island of New Zealand ( Gross *et al.*, 1979; Hultén & Fries, 1986; POWO, 2024).

**Figure 1.**
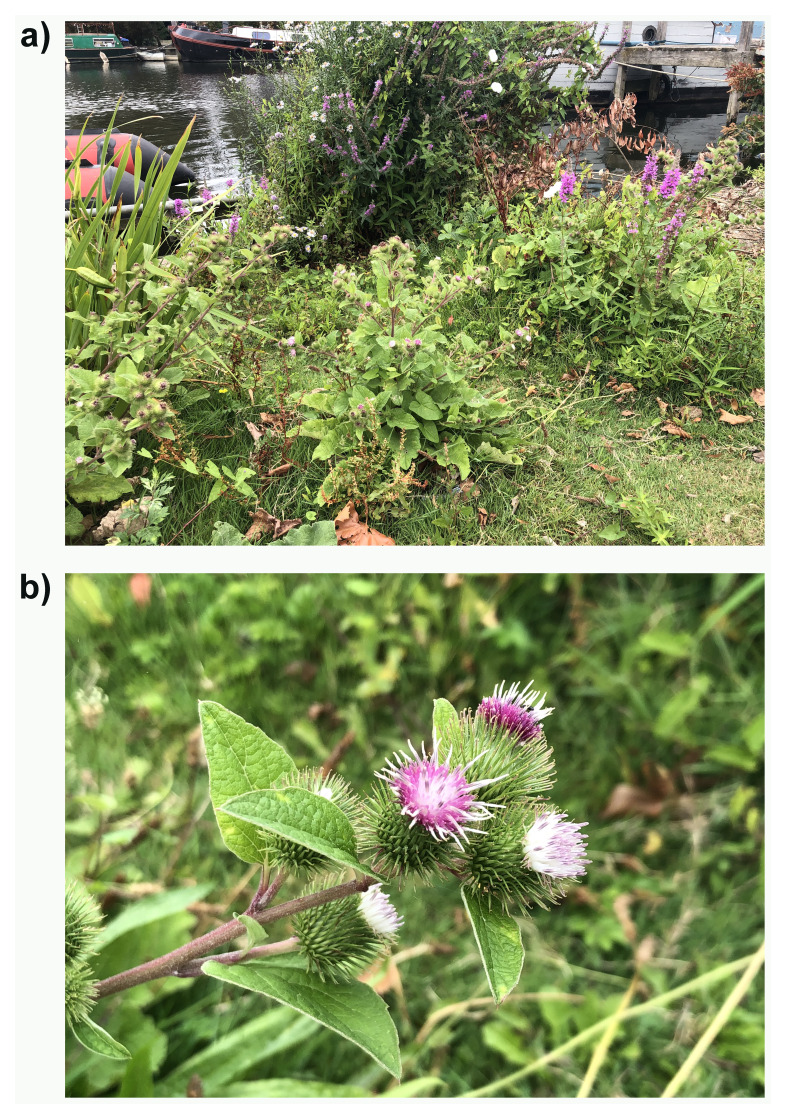
Photographs of the *Arctium minus* (daArcMinu1) specimen used for genome sequencing showing (a) the whole plant, and (b) a close up of one of the flowering stems.

The life cycle of the plant spans two years; in the first year, it forms a basal rosette of leaves, while in the second year, it produces tall, branched flowering stems reaching heights of 1 to 2 metres ( Stace *et al.*, 2019). The flowering period extends from July to September, during which purple flowers, similar to that of thistles, appear ( Figure 1). When dry, these flowers turn brown and form ‘burrs’ that cling to animal fur and clothing. This mechanism of seed dispersal inspired technological innovations such as Velcro, which utilises the hook and loop fastening mechanism observed and invented by George de Mestral, when he saw seed heads of burdock entangled in his dog’s fur ( Christenhusz *et al.*, 2017). The species forms an important food source for many species of Lepidoptera in Britain.

*Arctium minus* has been valued for both its culinary and medicinal uses. The roots of the plant, rich in inulin, have been consumed as a vegetable in some cultures (tasting like a cross between sweet chestnut and parsnip). Inulin is a soluble dietary fibre found in a variety of plants, and it is known for its prebiotic effects, promoting the growth of beneficial gut bacteria ( Moro & Clerici, 2021). In traditional medicine, the plant was utilised for its purported detoxifying properties ( Chevallier, 1996), and its extracted seed oil is rich in fatty acids and phytosterols. In modern-day Britain, most people will only be aware of the use of this species in the production of ‘burdock beer’, a traditional British soft drink, made from the roots of this and related species, often mixed with dandelion ( *Taraxacum* L.) roots, to create the well-known beverage ‘dandelion and burdock’, consumed since the Middle Ages. Originally a type of mead, it is now a carbonated soft drink ( Lewis-Stempel, 2010).

The genus name *Arctium* is derived from the Ancient Greek Ἄρκτον (romanised as Arctus), meaning bear, but it was also the name of a centaur in Greek mythology. It likely refers to the rough, bristly appearance of the burrs and perhaps the toughness of the plant. The Latin species epithet “minus”, small, denotes the relatively small size compared to the greater burdock ( *A. lappa* L.). The taxonomic history of *A. minus* has been chequered, and it has sometimes been treated as a variety or subspecies of *A. lappa* or *A. nemorosum* Lej. The species is variable, and identification can sometimes be difficult as it can closely resemble other species of the genus, especially wood burdock ( *A. nemorosum;* Stace *et al.*, 2019).

While *A. minus* has been reported to be a diploid with either a chromosome number of 2 *n* = 2 *x* = 32 or 36, all UK material counted to date shows it to be 2 *n* = 36 ( Stace *et al.*, 2019), and the previous reports of 2 *n* = 32 are now considered to erroneous ( Gross *et al.*, 1980). Species of the genus *Arctium* are known to hybridise ( Wang *et al.*, 2019), and this has been documented from Britain and Ireland. As a result, polyploidy is frequent in related species, contributing to further identification challenges and taxonomic debates. Hybrids such as *Arctium × nothum* (Ruhmer) J.Weiss (a hybrid between *A. minus* and *A. lappa*) and *Arctium × mixtum* (Simonk.) Nyman (a hybrid between *A. minus* and *A. tomentosum* Mill., woolly burdock) exhibit triploid chromosome counts (2 *n* = 3 *x* = 54), likely arising from unreduced gametes from one parent. These hybrid zones, primarily areas where the ranges of the parent species overlap, are typically in disturbed habitats that provide suitable conditions for both species to co-occur. The presence of hybrids indicates active gene flow between the species, resulting in intermediate forms, but this typically leads to reduced fertility resulting in low persistence of these cytotypes.

Here, we present the first chromosome-level *A. minus* genome, which we anticipate will help in understanding the taxonomic diversity of the genus, including hybrid evolution, and facilitate comparative genomic studies to uncover evolutionary and functional genomic insights. Secondly, as a highly tolerant species, this genome can help to enhance our understanding of the genetic basis of adaptability and resilience in diverse environments. In addition, this provides the opportunity to explore genes involved in the synthesis of medicinal compounds like inulin and antimicrobial agents.

### Genome sequence report

The genome of a specimen of *Arctium minus* ( Figure 1) was sequenced using Pacific Biosciences single-molecule HiFi long reads, generating a total of 72.51 Gb (gigabases) from 5.53 million reads, providing approximately 24-fold coverage. Using flow cytometry, the genome size (1C-value) was estimated to be 2.11 pg, equivalent to 2,070 Mb. Primary assembly contigs were scaffolded with chromosome conformation Hi-C data, which produced 213.23 Gb from 1,412.15 million reads, yielding an approximate coverage of 112-fold. Specimen and sequencing information is summarised in Table 1.

**Table 1.** Specimen and sequencing data for *Arctium minus*.

Project information			
**Study title**	Arctium minus		
**Umbrella BioProject**	PRJEB53860		
**Species**	*Arctium minus*		
**BioSample**	SAMEA7521931		
**NCBI taxonomy ID**	143172		
Specimen information			
**Technology**	**ToLID**	**BioSample accession**	**Organism part**
**PacBio long read sequencing**	daArcMinu1	SAMEA7521964	leaf
**Hi-C sequencing**	daArcMinu1	SAMEA7521962	leaf
**RNA sequencing**	daArcMinu1	SAMEA7521959	flower
Sequencing information			
**Platform**	**Run accession**	**Read count**	**Base count (Gb)**
**Hi-C Illumina NovaSeq 6000**	ERR9881701	1.41e+09	213.23
**PacBio Sequel IIe**	ERR9902008	9.86e+05	14.05
**PacBio Sequel IIe**	ERR9902011	1.90e+06	23.34
**PacBio Sequel IIe**	ERR9902009	9.75e+05	13.81
**PacBio Sequel IIe**	ERR9902010	1.67e+06	21.32
**RNA Illumina NovaSeq 6000**	ERR10378020	5.62e+07	8.48
**RNA Illumina NovaSeq 6000**	ERR10378019	5.89e+07	8.9

**Figure 2.**
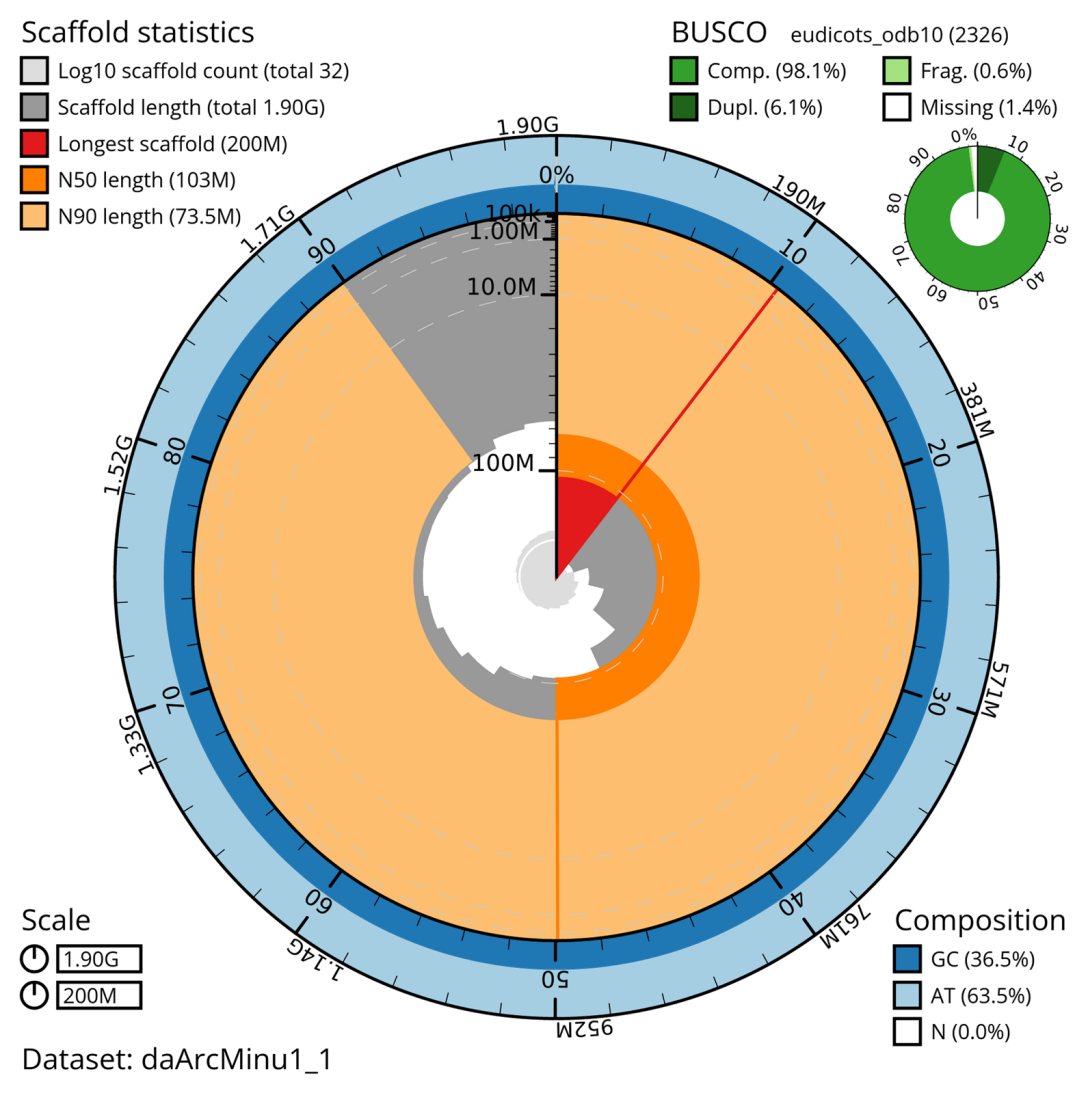
Genome assembly of *Arctium minus*, daArcMinu1.1: metrics. The BlobToolKit snail plot shows N50 metrics and BUSCO gene completeness. The main plot is divided into 1,000 size-ordered bins around the circumference with each bin representing 0.1% of the 1,903,528,901 bp assembly. The distribution of scaffold lengths is shown in dark grey with the plot radius scaled to the longest scaffold present in the assembly (199,739,593 bp, shown in red). Orange and pale-orange arcs show the N50 and N90 scaffold lengths (103,461,716 and 73,472,875 bp), respectively. The pale grey spiral shows the cumulative scaffold count on a log scale with white scale lines showing successive orders of magnitude. The blue and pale-blue area around the outside of the plot shows the distribution of GC, AT and N percentages in the same bins as the inner plot. A summary of complete, fragmented, duplicated and missing BUSCO genes in the eudicots_odb10 set is shown in the top right. An interactive version of this figure is available at https://blobtoolkit.genomehubs.org/view/daArcMinu1_1/dataset/daArcMinu1_1/snail.

**Figure 3.**
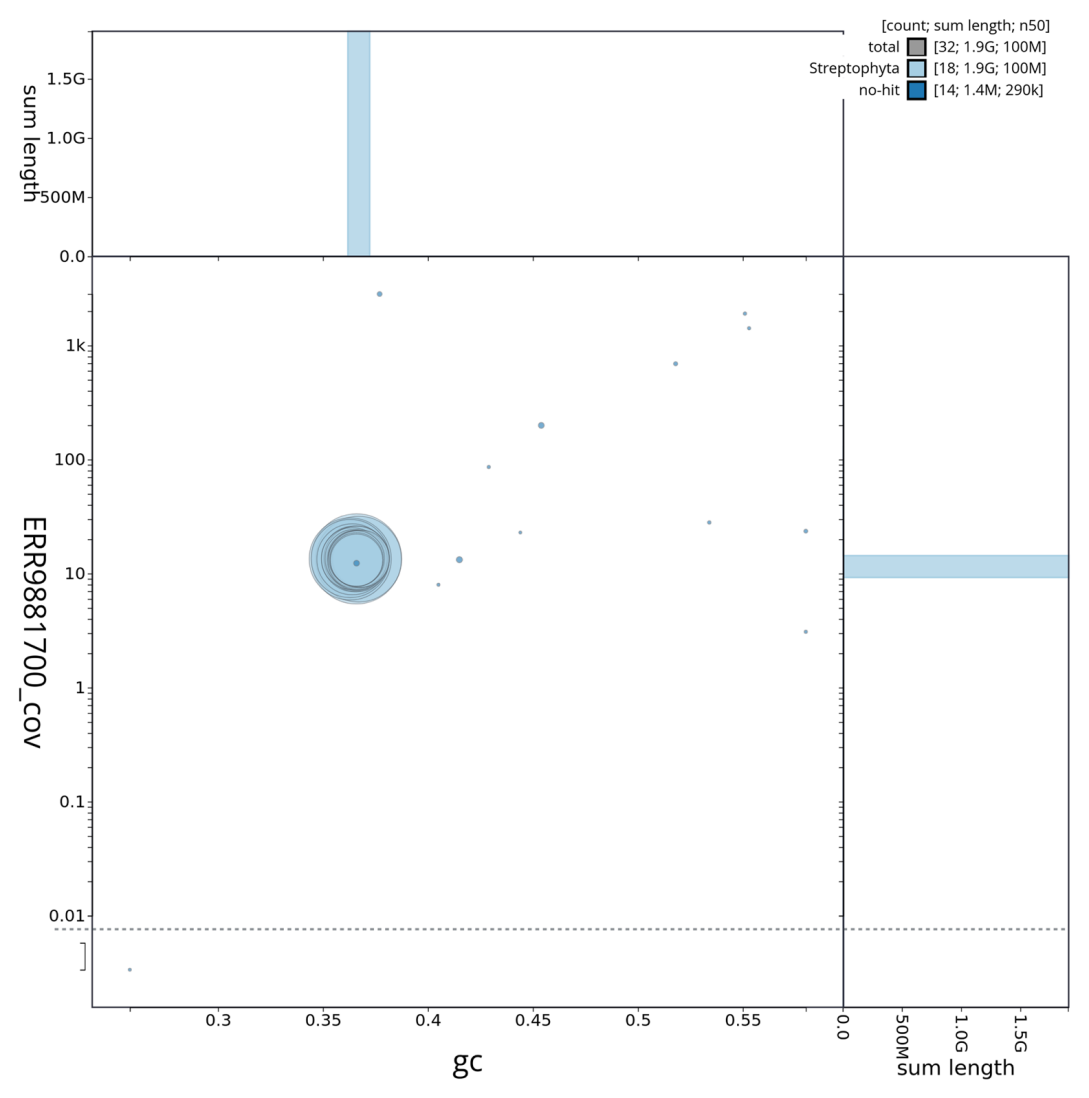
Blob plot of base coverage against GC proportion for sequences in the assembly daArcMinu1.1 Sequences are coloured by phylum. Circles are sized in proportion to sequence length. Histograms show the distribution of sequence length sum along each axis. An interactive version of this figure is available at https://blobtoolkit.genomehubs.org/view/daArcMinu1_1/dataset/daArcMinu1_1/blob.

**Figure 4.**
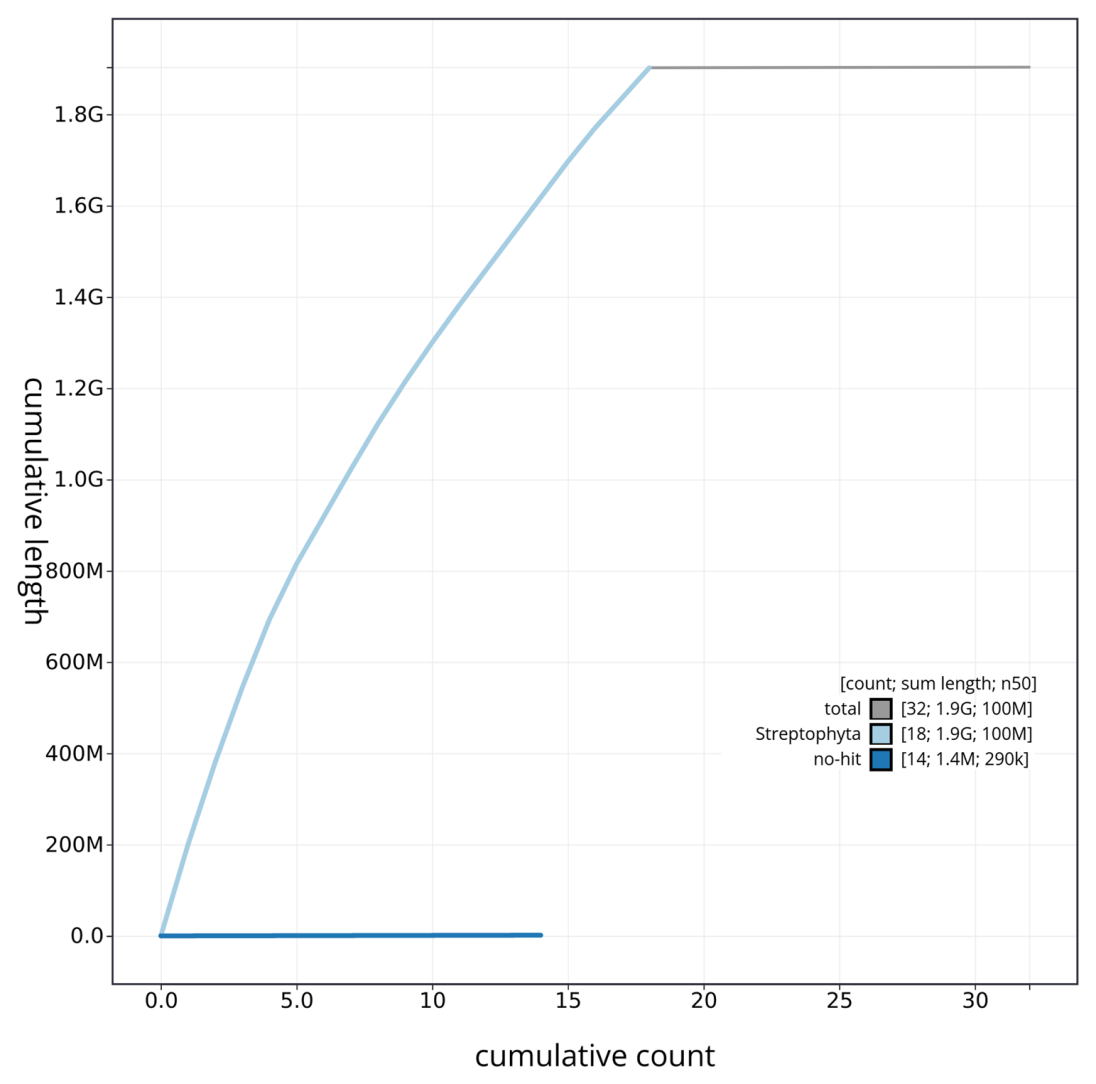
Genome assembly of *Arctium minus* daArcMinu1.1: BlobToolKit cumulative sequence plot. The grey line shows cumulative length for all sequences. Coloured lines show cumulative lengths of sequences assigned to each phylum using the buscogenes taxrule. An interactive version of this figure is available at https://blobtoolkit.genomehubs.org/view/daArcMinu1_1/dataset/daArcMinu1_1/cumulative.

**Figure 5.**
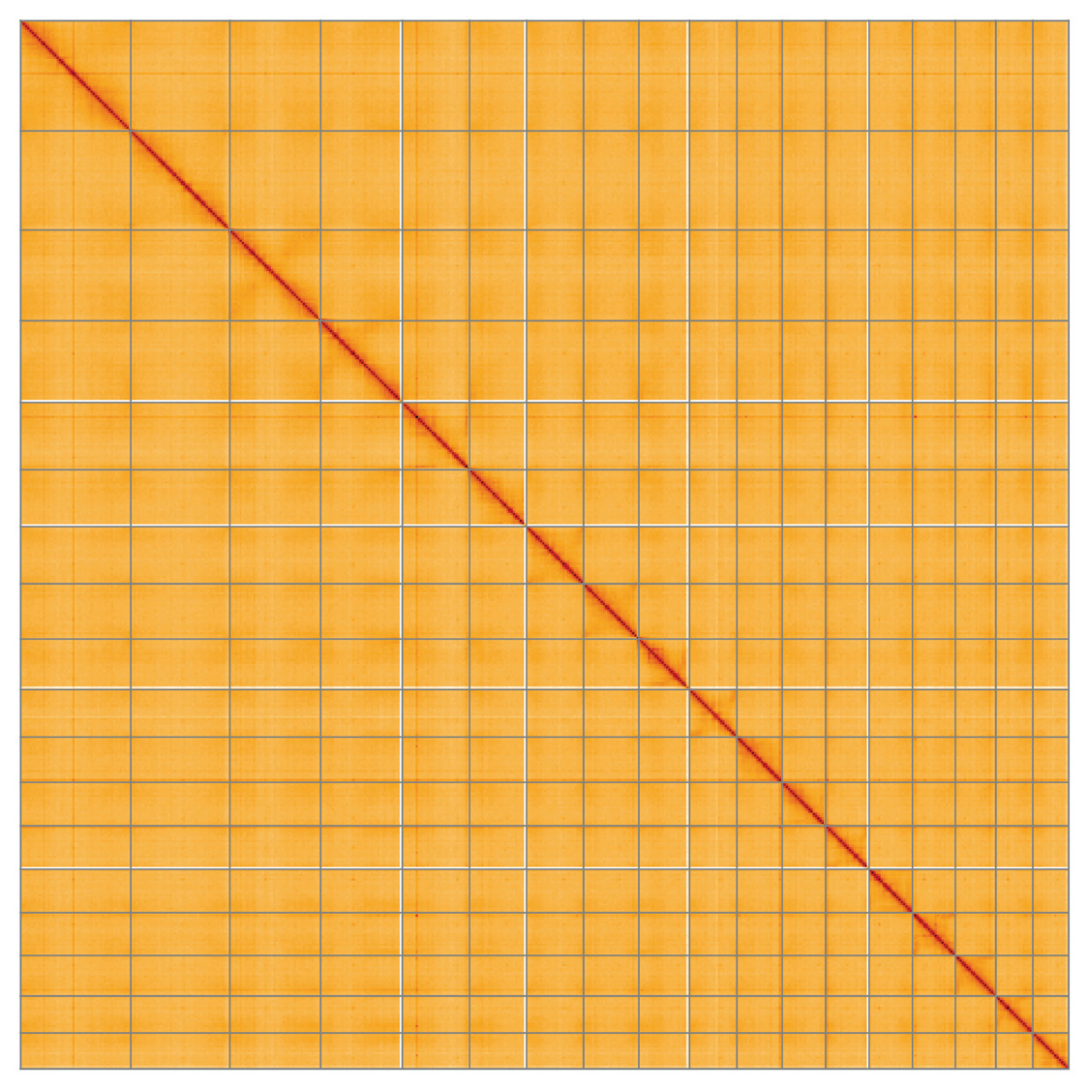
Genome assembly of *Arctium minus*, daArcMinu1.1: Hi-C contact map of the daArcMinu1.1 assembly, visualised using HiGlass. Chromosomes are shown in order of size from left to right and top to bottom. An interactive version of this figure may be viewed at https://genome-note-higlass.tol.sanger.ac.uk/l/?d=Ej0r1nfwSweTrM1Vac65yg.

Manual assembly curation corrected two missing joins or mis-joins and two haplotypic duplications. The final assembly has a total length of 1,903.10 Mb in 30 sequence scaffolds with a scaffold N50 of 103.5 Mb ( Table 2) with 14 gaps. The snail plot in Figure 2 summarises the assembly statistics, while the blob plot in Figure 3 shows the distribution of assembly scaffolds by GC proportion and coverage. The cumulative assembly plot in Figure 4 shows curves for subsets of scaffolds assigned to different phyla. Most (99.92%) of the assembly sequence was assigned to 18 chromosomal-level scaffolds. Chromosome-scale scaffolds confirmed by the Hi-C data are named in order of size ( Figure 5; Table 3). While not fully phased, the assembly deposited is of one haplotype. Contigs corresponding to the second haplotype have also been deposited. The mitochondrial and plastid genomes were also assembled and can be found as contigs within the multifasta file of the genome submission.

**Table 2.** Genome assembly data for *Arctium minus*, daArcMinu1.1.

Genome assembly		
Assembly name	daArcMinu1.1	
Assembly accession	GCA_954870635.1	
*Accession of alternate haplotype*	*GCA_954871535.1*	
Span (Mb)	1,903.10	
Number of contigs	46	
Contig N50 length (Mb)	80.9	
Number of scaffolds	30	
Scaffold N50 length (Mb)	103.5	
Longest scaffold (Mb)	199.74	
Assembly metrics *		*Benchmark*
Consensus quality (QV)	60.7	*≥ 50*
*k*-mer completeness	100.0%	*≥ 95%*
BUSCO **	C:98.1%[S:92.0%,D:6.1%], F:0.6%,M:1.4%,n:2,326	*C ≥ 95%*
Percentage of assembly mapped to chromosomes	99.92%	*≥ 95%*
Organelles	Mitochondrial genome: 312.58 kb; plastid genome: 152.71 kb	*complete single alleles*
Genome annotation at Ensembl		
Number of protein-coding genes	27,734	
Number of non-coding genes	10,938	
Number of gene transcripts	52,022	

* Assembly metric benchmarks are adapted from column VGP-2020 of “Table 1: Proposed standards and metrics for defining genome assembly quality” from Rhie *et al.* (2021). ** BUSCO scores based on the eudicots_odb10 BUSCO set using version 5.4.3. C = complete [S = single copy, D = duplicated], F = fragmented, M = missing, n = number of orthologues in comparison. A full set of BUSCO scores is available at https://blobtoolkit.genomehubs.org/view/daArcMinu1_1/dataset/daArcMinu1_1/busco.

**Table 3.** Chromosomal pseudomolecules in the genome assembly of *Arctium minus*, daArcMinu1.

INSDC accession	Name	Length (Mb)	GC%
OX941080.1	1	199.74	36.5
OX941081.1	2	179.92	36.5
OX941082.1	3	164.54	36.5
OX941083.1	4	148.83	36.5
OX941084.1	5	121.84	36.5
OX941085.1	6	103.58	36.5
OX941086.1	7	103.46	36.5
OX941087.1	8	100.14	36.5
OX941088.1	9	91.98	36.5
OX941089.1	10	86.24	36.5
OX941090.1	11	81.9	36.5
OX941091.1	12	79.15	36.5
OX941092.1	13	78.86	36.5
OX941093.1	14	78.65	36.5
OX941094.1	15	77.81	37.0
OX941095.1	16	73.47	36.5
OX941096.1	17	67.44	36.5
OX941097.1	18	64.56	36.5
OX941098.1	MT	0.31	45.5
OX941099.1	Pltd	0.15	37.5

The estimated Quality Value (QV) of the final assembly is 60.7 with *k*-mer completeness of 100.0%, and the assembly has a BUSCO v5.4.3 completeness of 98.1% (single = 92.0%, duplicated = 6.1%), using the eudicots_odb10 reference set ( *n* = 2,326).

Metadata for specimens, BOLD barcode results, spectra estimates, sequencing runs, contaminants and pre-curation assembly statistics are given at https://links.tol.sanger.ac.uk/species/143172.

**Genome annotation report**

The *Arctium minus* genome assembly (GCA_954870635.1) was annotated at the European Bioinformatics Institute (EBI) on Ensembl Rapid Release. The resulting annotation includes 52,022 transcribed mRNAs from 27,734 protein-coding and 10,938 non-coding genes ( Table 2; https://rapid.ensembl.org/Arctium_minus_GCA_954870635.1/Info/Index). The average transcript length is 3,940.95. There are 1.35 coding transcripts per gene and 4.83 exons per transcript.

## Methods

### Sample acquisition, DNA barcoding and genome size estimation

A specimen of *Arctium minus* (specimen ID KDTOL10022, ToLID daArcMinu1) was collected from Canbury Gardens, Kingston Upon Thames, Surrey, UK (latitude 51.42, longitude –0.31) on 2020-08-06. The specimen was collected and identified by Maarten Christenhusz (Royal Botanic Gardens, Kew) and preserved by freezing at –80°C. The herbarium voucher associated with the sequenced plant is M. Christenhusz 9019 and is deposited in the herbarium of RBG Kew (K) (K001400639).

The initial species identification was verified by an additional DNA barcoding process following the framework developed by Twyford *et al.* (2024). Part of the plant specimen was preserved in silica gel desiccant ( Chase & Hills, 1991). DNA was extracted from the dried specimen, then PCR was used to amplify standard barcode regions. The resulting amplicons were sequenced and compared to public sequence databases including GenBank and the Barcode of Life Database (BOLD). The barcode sequences for this specimen are available on BOLD ( Ratnasingham & Hebert, 2007). Following whole genome sequence generation, DNA barcodes were also used alongside the initial barcoding data for sample tracking through the genome production pipeline at the Wellcome Sanger Institute ( Twyford *et al.*, 2024). The standard operating procedures for the Darwin Tree of Life barcoding have been deposited on protocols.io ( Beasley *et al.*, 2023).

The genome size was estimated by flow cytometry using the fluorochrome propidium iodide and following the ‘one-step’ method as outlined in Pellicer *et al.* (2021). For this species, the General Purpose Buffer (GPB) supplemented with 3% PVP and 0.08% (v/v) beta-mercaptoethanol was used for isolation of nuclei ( Loureiro *et al.*, 2007), and the internal calibration standard was *Solanum lycopersicum* ‘Stupiké polní rané’ with an assumed 1C-value of 968 Mb ( Doležel *et al.*, 2007).

### Nucleic acid extraction

The workflow for high molecular weight (HMW) DNA extraction at the Wellcome Sanger Institute (WSI) Tree of Life Core Laboratory includes a sequence of core procedures: sample preparation; sample homogenisation, DNA extraction, fragmentation, and clean-up. Detailed protocols are available on protocols.io ( Denton *et al.*, 2023). The daArcMinu1 sample was weighed and dissected on dry ice ( Jay *et al.*, 2023). For sample homogenisation, leaf tissue was cryogenically disrupted using the Covaris cryoPREP ^®^ Automated Dry Pulverizer ( Narváez-Gómez *et al.*, 2023).

HMW DNA was extracted using the Automated Plant MagAttract v2 protocol ( Todorovic *et al.*, 2023). HMW DNA was sheared into an average fragment size of 12–20 kb in a Megaruptor 3 system ( Bates *et al.*, 2023). Sheared DNA was purified by solid-phase reversible immobilisation, using AMPure PB beads to eliminate shorter fragments and concentrate the DNA ( Strickland *et al.*, 2023). The concentration of the sheared and purified DNA was assessed using a Nanodrop spectrophotometer and Qubit Fluorometer and Qubit dsDNA High Sensitivity Assay kit. Fragment size distribution was evaluated by running the sample on the FemtoPulse system.

RNA was extracted from flower tissue of daArcMinu1 in the Tree of Life Laboratory at the WSI using the RNA Extraction: Automated MagMax™ *mir*Vana protocol ( do Amaral *et al.*, 2023). The RNA concentration was assessed using a Nanodrop spectrophotometer and a Qubit Fluorometer using the Qubit RNA Broad-Range Assay kit. Analysis of the integrity of the RNA was done using the Agilent RNA 6000 Pico Kit and Eukaryotic Total RNA assay.

### Hi-C preparation

Leaf tissue of daArcMinu1 was processed at the WSI Scientific Operations core, using the Arima-HiC v2 kit. Tissue was finely ground using cryoPREP and then subjected to nuclei isolation using a modified protocol of the Qiagen QProteome Kit. After isolation, the nuclei were fixed, and the DNA crosslinked using 37% formaldehyde solution. The crosslinked DNA was then digested using the restriction enzyme master mix. The 5’-overhangs were then filled in and labelled with biotinylated nucleotides and proximally ligated. An overnight incubation was carried out for enzymes to digest remaining proteins and for crosslinks to reverse. A clean up was performed with SPRIselect beads prior to library preparation. DNA concentration was quantified using the Qubit Fluorometer v2.0 and Qubit HS Assay Kit according to the manufacturer’s instructions.

### Library preparation and sequencing

Library preparation and sequencing was performed at the WSI Scientific Operations core. Pacific Biosciences HiFi circular consensus DNA sequencing libraries were prepared using the PacBio Express Template Preparation Kit v2.0 (Pacific Biosciences, California, USA) as per the manufacturer's instructions. The kit includes the reagents required for removal of single-strand overhangs, DNA damage repair, end repair/A-tailing, adapter ligation, and nuclease treatment. Library preparation also included a library purification step using 0.8X AMPure PB beads (Pacific Biosciences, California, USA) and a size selection step to remove templates <3 kb using AMPure PB modified SPRI. Samples were sequenced using the Sequel IIe system (Pacific Biosciences, California, USA). The concentration of the library loaded onto the Sequel IIe was within the manufacturer’s recommended loading concentration range of 40–100 pM. The SMRT link software, a PacBio web-based end-to-end workflow manager, was used to set-up and monitor the run, as well as perform primary and secondary analyses of the data upon completion.

Poly(A) RNA-Seq libraries were constructed using the NEB Ultra II RNA Library Prep kit following manufacturer’s instructions. RNA sequencing was performed on the Illumina NovaSeq 6000 instrument.

For Hi-C library preparation, DNA was fragmented to a size of 400 to 600 bp using a Covaris E220 sonicator. The DNA was then enriched, barcoded, and amplified using the NEBNext Ultra II DNA Library Prep Kit, following manufacturers’ instructions. The Hi-C sequencing was performed using paired-end sequencing with a read length of 150 bp on an Illumina NovaSeq 6000.

### Genome assembly, curation and evaluation

***Assembly***

The original assembly of HiFi reads was performed using Hifiasm ( Cheng *et al.*, 2021) with the --primary option. Haplotypic duplications were identified and removed with purge_dups ( Guan *et al.*, 2020). Hi-C reads were further mapped with bwa-mem2 ( Vasimuddin *et al.*, 2019) to the primary contigs, which were further scaffolded using the provided Hi-C data ( Rao *et al.*, 2014) in YaHS ( Zhou *et al.*, 2023) using the --break option. Scaffolded assemblies were evaluated using Gfastats ( Formenti *et al.*, 2022), BUSCO ( Manni *et al.*, 2021) and MERQURY.FK ( Rhie *et al.*, 2020).

The organelle genomes were assembled using MBG ( Rautiainen & Marschall, 2021) from PacBio HiFi reads mapping to related genomes. A representative circular sequence was selected for each from the graph based on read coverage.

***Curation***

The assembly was decontaminated using the Assembly Screen for Cobionts and Contaminants (ASCC) pipeline (article in preparation). Manual curation was primarily conducted using PretextView ( Harry, 2022), with additional insights provided by JBrowse2 ( Diesh *et al.*, 2023) and HiGlass ( Kerpedjiev *et al.*, 2018). Scaffolds were visually inspected and corrected as described by Howe *et al.*, (2021). Any identified contamination, missed joins, and mis-joins were corrected, and duplicate sequences were tagged and removed. The process is documented at https://gitlab.com/wtsi-grit/rapid-curation (article in preparation).

***Evaluation of final assembly***

A Hi-C map for the final assembly was produced using bwa-mem2 ( Vasimuddin *et al.*, 2019) in the Cooler file format ( Abdennur & Mirny, 2020). To assess the assembly metrics, the *k*-mer completeness and QV consensus quality values were calculated in Merqury ( Rhie *et al.*, 2020). This work was done using the “sanger-tol/readmapping” ( Surana *et al.*, 2023a) and “sanger-tol/genomenote” ( Surana *et al.*, 2023b) pipelines. The genome evaluation pipelines were developed using nf-core tooling ( Ewels *et al.*, 2020) and MultiQC ( Ewels *et al.*, 2016), relying on the Conda package manager, the Bioconda initiative ( Grüning *et al.*, 2018), the Biocontainers infrastructure ( da Veiga Leprevost *et al.*, 2017), as well as the Docker ( Merkel, 2014) and Singularity ( Kurtzer *et al.*, 2017) containerisation solutions.

The genome was also analysed within the BlobToolKit environment ( Challis *et al.*, 2020) and BUSCO scores ( Manni *et al.*, 2021) were calculated.

Table 4 contains a list of relevant software tool versions and sources.

**Table 4.** Software tools: versions and sources.

Software tool	Version	Source
BlobToolKit	4.1.7	https://github.com/blobtoolkit/blobtoolkit
BUSCO	5.3.2	https://gitlab.com/ezlab/busco
bwa-mem2	2.2.1	https://github.com/bwa-mem2/bwa-mem2
Cooler	0.8.11	https://github.com/open2c/cooler
Gfastats	1.3.6	https://github.com/vgl-hub/gfastats
Hifiasm	0.16.1-r375	https://github.com/chhylp123/hifiasm
HiGlass	1.11.6	https://github.com/higlass/higlass
MBG	-	https://github.com/maickrau/MBG
Merqury	MerquryFK	https://github.com/thegenemyers/MERQURY.FK
PretextView	0.2	https://github.com/wtsi-hpag/PretextView
purge_dups	1.2.3	https://github.com/dfguan/purge_dups
sanger-tol/genomenote	v1.0	https://github.com/sanger-tol/genomenote
sanger-tol/readmapping	1.1.0	https://github.com/sanger-tol/readmapping/tree/1.1.0
YaHS	yahs-1.1.91eebc2	https://github.com/c-zhou/yahs

### Wellcome Sanger Institute – Legal and Governance

The materials that have contributed to this genome note have been supplied by a Darwin Tree of Life Partner. The submission of materials by a Darwin Tree of Life Partner is subject to the **‘Darwin Tree of Life Project Sampling Code of Practice’**, which can be found in full on the Darwin Tree of Life website here. By agreeing with and signing up to the Sampling Code of Practice, the Darwin Tree of Life Partner agrees they will meet the legal and ethical requirements and standards set out within this document in respect of all samples acquired for, and supplied to, the Darwin Tree of Life Project.

Further, the Wellcome Sanger Institute employs a process whereby due diligence is carried out proportionate to the nature of the materials themselves, and the circumstances under which they have been/are to be collected and provided for use. The purpose of this is to address and mitigate any potential legal and/or ethical implications of receipt and use of the materials as part of the research project, and to ensure that in doing so we align with best practice wherever possible. The overarching areas of consideration are:

•   Ethical review of provenance and sourcing of the material

•   Legality of collection, transfer and use (national and international)

Each transfer of samples is further undertaken according to a Research Collaboration Agreement or Material Transfer Agreement entered into by the Darwin Tree of Life Partner, Genome Research Limited (operating as the Wellcome Sanger Institute), and in some circumstances other Darwin Tree of Life collaborators.

## Data Availability

European Nucleotide Archive: *Arctium minus*. Accession number PRJEB53860; https://identifiers.org/ena.embl/PRJEB53860 ( Wellcome Sanger Institute, 2023). The genome sequence is released openly for reuse. The *Arctium minus* genome sequencing initiative is part of the Darwin Tree of Life (DToL) project. All raw sequence data and the assembly have been deposited in INSDC databases. Raw data and assembly accession identifiers are reported in Table 1. Members of the Royal Botanic Gardens Kew Genome Acquisition Lab are listed here: https://doi.org/10.5281/zenodo.12625079. Members of the Plant Genome Sizing collective are listed here: https://doi.org/10.5281/zenodo.7994306. Members of the Darwin Tree of Life Barcoding collective are listed here: https://doi.org/10.5281/zenodo.12158331. Members of the Wellcome Sanger Institute Tree of Life Management, Samples and Laboratory team are listed here: https://doi.org/10.5281/zenodo.12162482. Members of Wellcome Sanger Institute Scientific Operations: Sequencing Operations are listed here: https://doi.org/10.5281/zenodo.12165051. Members of the Wellcome Sanger Institute Tree of Life Core Informatics team are listed here: https://doi.org/10.5281/zenodo.12160324. Members of the Tree of Life Core Informatics collective are listed here: https://doi.org/10.5281/zenodo.12205391. Members of the Darwin Tree of Life Consortium are listed here: https://doi.org/10.5281/zenodo.4783558.
