# Supplementary material for: The genome sequence of lesser burdock, Arctium minus (Hill) Bernh. (Asteraceae)
Source: Wellcome Open Res. 2024 Oct 15;9:589. [Version 1] doi: 10.12688/wellcomeopenres.23160.1 (PMC11574341; doi:10.12688/wellcomeopenres.23160.1)
Supplement: Peer review history

# Reviewer report: Reviewer response for version 1

**DOI:** 10.21956/wellcomeopenres.25504.r109244

**Published:** 2024-11-28

**Authors:** Eric González-Segovia

**Affiliations:** 1 University of British Columbia, British Columbia, Canada

The article discusses the *Arctium minus* genome assembly, providing a brief overview of the species' biology and its uses. The methods for the specimen collection, DNA extraction, HiFi and Hi-C sequencing as well the genome assembly are well detailed and clearly described.

 I have just a minor comment regarding these lines: *"While not fully phased, the assembly deposited is of one haplotype. Contigs corresponding to the second haplotype have also been deposited."*

 From my understanding, the authors employed a primary/alternate assembly approach. In this approach, the primary assembly consists of a mixture of both haplotypes, where the best and longer contigs are selected. The alternate assembly contains contigs for heterozygous regions not included in the primary assembly. Given this, I believe it is inaccurate to state that *"the assembly deposited is of one haplotype"* since the primary assembly is a mixture of haplotypes rather than a single haplotype. Similarly, the alternate assembly does not consist solely of contigs from a single haplotype. Instead, they could simply state that the chromosome-level assembly was created using the primary assembly and that the alternate assembly is also available.

 Other than this minor comment, the article is well written, and everything was clear to me. However, I do not possess expertise in this species to verify the accuracy of the biological details provided.

Are sufficient details of methods and materials provided to allow replication by others?

Yes

Is the rationale for creating the dataset(s) clearly described?

Yes

Are the datasets clearly presented in a useable and accessible format?

Yes

Are the protocols appropriate and is the work technically sound?

Yes

Reviewer Expertise:

Genetics, genomics and local adaptation.

I confirm that I have read this submission and believe that I have an appropriate level of expertise to confirm that it is of an acceptable scientific standard.

## References

[1] : Whole genomes from the extinct Xerces Blue butterfly can help identify declining insect species. *Elife* .2024;12: 10.7554/eLife.87928 10.7554/eLife.87928 39365295 PMC11466284

# Reviewer report: Reviewer response for version 1

**DOI:** 10.21956/wellcomeopenres.25504.r109240

**Published:** 2024-11-18

**Authors:** Ayoob Obaid Alfalahi

**Affiliations:** 1 University of Anbar, Ramadi, Al Anbar Governorate, Iraq

In this article “The genome sequence of lesser burdock, *Arctium minus* (Hill) Bernh. (Asteraceae)”, authors produced high-quality genome assembly for diploid *Arctium minus* (Hill) Bernh using DNA barcoding, HiFi, and Hi-C map techniques. The results revealed a genome size of 2,070 mb scaffolded into 18 chromosomal pseudomolecules, along with mitochondrial and plastid genome assemblies. The results are really interesting and will provide insights into chromosomal evolution of this biennial species, in addition to better understanding on adaptation, genomic, and proteomic levels.

 The following notes may be considered:

1. In Figure 1, a) more than one plant species are presented in the provided photo which is a bit confusing, hence a close shot indicating only the targeted plant species ( *Arctium minus*) may be provided. Also, it would be better to include photo showing all the plant parts (root, stem, leaves, flowers and maybe fruits), this will be more descriptive and informative, especially in such specialized studies.   
2. The collection stage of plant sample should be determined, however, full blooming stage is preferred. Furthermore, in Table 1, authors pointed to different organism parts used for molecular analysis (leaf and flower), are these different parts from one plant sample!
3. Authors missed some relevant up-to-date references that can really support the presented information about *Arctium minus* (e.g https://doi.org/10.1007/s11130-024-01175-w).
4. The introduction included long stories without relevant references supporting these claims, especially the last section describing the polyploidy phenomenon in *Arctium*.
5. Links for the used software should be provided. The manufactured company, address and origin should be stated in all methodology part not only for Library preparation and sequencing work.

Are sufficient details of methods and materials provided to allow replication by others?

Yes

Is the rationale for creating the dataset(s) clearly described?

Yes

Are the datasets clearly presented in a useable and accessible format?

Yes

Are the protocols appropriate and is the work technically sound?

Yes

Reviewer Expertise:

Plant Biotechnology

I confirm that I have read this submission and believe that I have an appropriate level of expertise to confirm that it is of an acceptable scientific standard.

# Reviewer report: Reviewer response for version 1

**DOI:** 10.21956/wellcomeopenres.25504.r105938

**Published:** 2024-11-05

**Authors:** Norma Paniego

**Affiliations:** 1 Instituto Nacional de Tecnología Agropecuaria; Consejo Nacional de Investigaciones Científicas y Técnicas, Instituto de Agrobiotecnología y Biología Molecular, Hurlingham, Buenos Aires, Argentina

In this Data Note, the authors present a chromosome-level genome assembly of *Arctium minus*, a biennial herbaceous plant of the Asteraceae family, using a combination of PACBIO HiFi and HiC Illumina sequencing technologies. *A. minu*s, like other members of the genus Arctium, is valued for its edible and medicinal properties. This species is native to Europe and Western Asia and is distributed in different regions worldwide due to its high adaptability to various soils and environments.

 This work provides information on the genome size of *A. minu*s, estimated at 2,070 Mb. The results are clearly described and are complemented by complete tables and figures. The figures have interactive versions that can be accessed via web links to understand the results better. The study provides a high-quality assembly of the nuclear genome into 18 chromosomes, together with genome annotations. The mitochondrial and plastid genomes are also presented. The methods applied are described in detail and linked to standardized protocols available on the protocol.io platform. In addition, the software used and the quality control metrics applied are clearly outlined and referenced. All data and metadata, including sequences, annotations, and herbarium specimens, are accessible in public repositories.

 The work is original, well written, and provides valuable information for the plant community interested in the Arctium family, as well as for groups studying genes involved in the synthesis of medicinal compounds. Furthermore, being a highly resilient species, this genome can contribute to the understanding of the genetic basis of plant adaptability and resilience in different environments. Finally, from a technological perspective, this work is interesting for research groups involved in whole genome sequencing, as it presents relevant methodological aspects, including robust quality control metrics applied at distinct stages of the process, and efficient forms for graphical visualization of the results.

**Minor comments:**

**I** suggest adding in the Background section, the references to genome assembly of *A lappa* (1, 2) and the multi-omic database for *Arcticum sp*. (3). In the Results section, I note an inconsistency in the reporting of genome coverage for PACBIO and HiC sequencing. I recommend revising these values in relation to the total gigabases generated and the estimated genome size in this note.

Are sufficient details of methods and materials provided to allow replication by others?

Yes

Is the rationale for creating the dataset(s) clearly described?

Yes

Are the datasets clearly presented in a useable and accessible format?

Yes

Are the protocols appropriate and is the work technically sound?

Yes

Reviewer Expertise:

Plant genomics,  genomics of plant fungal disease resistance, plant genomics breeding

I confirm that I have read this submission and believe that I have an appropriate level of expertise to confirm that it is of an acceptable scientific standard.

## References

[1] : The complete mitochondrial genome ofArctium lappa (Campanulales, Asteraceae). *Mitochondrial DNA Part B* .2020;5(2) : 10.1080/23802359.2020.1749153 1722-1723 10.1080/23802359.2020.1749153

[2] : The first high-quality chromosomal genome assembly of a medicinal and edible plant Arctium lappa. *Mol Ecol Resour* .2022;22(4) : 10.1111/1755-0998.13547 1493-1507 10.1111/1755-0998.13547 34758188

[3] : The burdock database: a multi-omic database for Arctium lappa, a food and medicinal plant. *BMC Plant Biol* .2023;23(1) : 10.1186/s12870-023-04092-3 86 10.1186/s12870-023-04092-3 36759759 PMC9909940
